# Multi Band Gap Electronic Structure in CH_3_NH_3_PbI_3_

**DOI:** 10.1038/s41598-018-38023-2

**Published:** 2019-02-14

**Authors:** Khuong P. Ong, Shunnian Wu, Tien Hoa Nguyen, David J. Singh, Zhen Fan, Michael B. Sullivan, Cuong Dang

**Affiliations:** 1 Institute of High Performance Computing, Agency of Science, Technology and Research (A*STAR), 1 Fusionopolis Way, 138632 Singapore, Singapore; 2 Centre for OptoElectronics and Biophotonics (COEB), School of Electrical and Electronic Engineering, The Photonics Institute (TPI), Nanyang Technological University Singapore, 50 Nanyang Avenue, 639798 Singapore, Singapore; 3 Department of Physics and Astronomy, University of Missouri, Columbia, MO 65211-7010 USA; 4 Institute for Advanced Materials and Guangdong Provincial Key Laboratory of Optical Information Materials and Technology, South China Academy of Advanced Optoelectronics, South China Normal University, Guangzhou, 510006 China

## Abstract

Organo-lead halide perovskite solar cells represent a revolutionary shift in solar photovoltaics, introducing relatively soft defect containing semiconductors as materials with excellent charge collection for both electrons and holes. Although they are based on the nominally simple cubic perovskite structure, these compounds are in fact very complex. For example, in (CH_3_NH_3_)PbI_3_ the dynamics and ensuing structural fluctuations associated with the (CH_3_NH_3_)^+^ ions and the interplay with the electronic properties are still not fully understood, despite extensive study. Here, using ab-initio calculations, we show that at room and higher temperature, the rotation of CH_3_NH_3_ molecules can be viewed as effectively giving local structures that are cubic and tetragonal like from the point of view of the PbI_3_ framework, though in fact having lower symmetry. Both of these structures are locally polar, with sizable polarization, ~10 μC/cm^2^ due to the dipoles on the organic. They become energetically degenerate in the volume range, V ~ 250 Å^3^/f.u–265 Å^3^/f.u. We also find very significant dependence of the band gap on the local structure. This type of transition is analogous to a transition between two ferroelectric structures, where in-spite of strong electron phonon coupling, there is strong screening of charged defects which can lead to enhanced mobility and charge collection. The results provide insights into the enhanced light absorption near the band edge and good charge collection in this material.

## Introduction

The organic-inorganic hybrid perovskites (OIHP) have emerged as an important new class of photovoltaics, exemplified by methylammonium lead iodide CH_3_NH_3_PbI_3_ (MAPbI_3_), Laboratory devices based on these have reported efficiency exceeding 21%^1^ which is comparable to or even higher than the performance of existing solar cell technologies. The pace of research and ensuing progress is also impressive since first report^2^ on perovskite photovoltaic whose progress has outshined those of other solar cell types in photovoltaic research. Many efforts have made to increase the efficiency of OIHP by chemically and structurally adjusting the band gap and other properties^3,4^. In addition, the specific chemical bonding of divalent group IV elements with halides has been invoked as a possible explanation for the excellent charge collection in these materials. Specifically, the defect tolerance has been associated with high dielectric constants, and therefore defect tolerance^4^, as discussed in terms of high Born charges on the Pb and halogen atoms in these and related halides^5–7^. There is therefore a strong interplay between the lattice structure and bonding of the PbI_3_ part of the unit cell and the charge collection. This is complicated by the symmetry lowering but presumably electronically inactive organic cation on the perovskite A-site.

In addition to the dielectric properties, important for charge collection in these defected materials, light absorption beginning with optimized band gaps is crucial. The band gap can be tuned in various ways, but it is important that the successful approaches are ones that give not only optimal band gaps, but good absorption near the band edge, without degradation of the charge collection^7–9^. Rashba band splitting due to the spin - orbit coupling has been suggested as a possible cause for the reduced recombination rate^10–12^. For the I4/mcm structure, Zheng *et al*.^10^ have shown that the spin forbidden transition between conduction band and valence band reduced the recombination rate; a similar phenomenon has also been unveiled by Etienne *et al*.^11^ who studied the dynamical origin of the Rashba effect. Experimentally, Wang *et al*., proved that a weak indirect band gap presents in MPbI_3_ as a result of Rashba splitting of the conduction band due to the spin-orbit coupling^12^. However, it is to be emphasized that the Rashba splitting depends on symmetry lowering, closely related to polar order.

The NH_3_CH_3_ (MA) molecules have been theoretically shown to play important roles in MAPbI_3_. Theoretical reports by Ong *et al*.^13,14^ revealed how the rotation of molecule CH_3_NH_3_ couples to the PbI_3_ host leading to effective structural phase changes, at least locally. By using the Van der Waals corrected Density Functional Theory, Motta *et al*.^15^ revealed that the rotation of orientation of CH_3_NH_3_ molecule in cubic MAPbI_3_ from [111] direction to [110] direction distorts the PbI_6_ octahedral cage and results in indirect band gap. Such an effect has been also investigated by Gao, *et al*.^16^. On the opposite, under the hydrostatic pressure, Wang *et al*.^12^ reported that the Rashba splitting is reduced due to a pressure induced reduction in local electric field around the Pb atom. The role of molecule rotation is excluded in that report. Experimentally at finite temperature, the CH_3_NH_3_ molecule is reported to orient randomly^17–19^, giving a net overall cubic centrosymmetric state. In the cubic phase, the nuclear magnetic resonance (NMR)^20^ showed that the MA cations reorient themselves with picosecond scale dynamics at high temperature but freeze at low temperatures. Importantly, it has been shown that that there are sizable effects on the optical properties associated with the dynamics of the MA from experiments. In particular, Quarti and co-workers^21^ does not abruptly change at the cubic-tetragonal phase transition, but gradually changes from 270–400 K, in spite of the phase transition at 327 K^22^. The temperature dynamical correlations between MA molecules near the phase transition remain to be fully elucidated, but near the transition it is likely that there is substantial local correlation between the MA orientations, and therefore on the time scale of optical and electronic processes it is likely that the behavior is influenced by local structural effects associated with this dynamics. Therefore, the influence of the orientation of molecule to the properties of hybrid perovskite still needs further investigation. In the present work we examine the magnitudes of local structural effects on the electronic and optical properties, using first principles calculations for ordered structures (see Methods).

Considering the PbI_3_ part of the structure (for the discussion, but not in the calculations, which necessarily include the full atomic structure, including the dipoles associated with the organic A-site, we describe the structure of the PbI_3_ with space groups that describe to a close approximation this part of the cell), the tetragonal phase I4mcm is stable only at V < 252 Å/f.u theoretically^14^, at higher volume the cubic Pm-3m and tetragonal P4mm phase are more stable. The tetragonal P4mm is obtained from cubic Pm-3m by deforming only one lattice constant along the c-axis, c ≠ a) and the tetragonal I4/mcm is a further deformation from P4mm by the rotation of octahedral PbI_6_ anti-phase tilt around the c-axis. Since solar cell is working at room temperature and higher, therefore our research mainly focuses on Pm-3m and P4mm structures. In this report, we study the impact of MA molecules to the efficiency of hybrid perovskite based solar cell. Our results show that the MA units easily rotate in PbI_6_ cuboctahedral, consistent with experiment, and these rotations are coupled significantly to the PbI_3_ lattice inducing structural changes from cubic Pm-3m to tetragonal P4mm and vice versa. This causes a momentum dependent splitting of energy band by Rashba effect due to the spin-orbit coupling, prevents the electron recombination, and induces a multi band gap electronic structure.

## Results and Discussion

**Figure 1:**
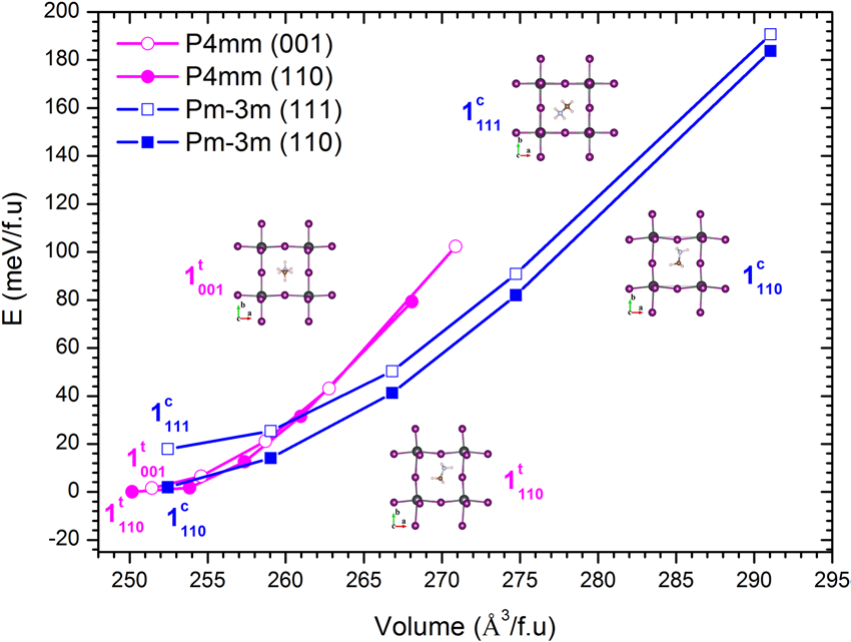
The energy-volume phase diagram of Pm-3m and P4mm with different MA orientations [111], [110] and [001]. The insets are crystal structures of MAPbI_3_ at different points ${1}_{110}^{t}$, ${1}_{001}^{t}$, ${1}_{110}^{c}$, and ${1}_{111}^{c}$, see text for details.

To study the impact of molecule rotation on the properties of MAPbI_3_, we study the influence of volume change to the rotation. We start with the most stable structure of P4mm with a = b = 6.32 Å, c = 6.31 Å^14^ and volume of V = 252 Å^3^, which is close to experimental data a = b = 6.312 Å, c = 6.316 Å, and V = 251.6 Å^3^ ^17^. The orientation of MA molecule is studied at three different directions: quasi-[111], quasi [001] and quasi-[110]. For the P4mm, at each of specific lattice constant a = b and specific orientation of MA molecule the lattice constant c is fully relaxed. Our study shows that the orientation of MA molecule along the quasi-[001] direction gives almost the same energy as the quasi-[110] direction, see Fig. 1.

The energy-volume phase diagram of structures P4mm and Pm-3m (for the PbI_3_ part of the cell) is given in Fig. 1. The result shows that at the same volume there is no preferred direction for MA molecules in tetragonal P4mm. On the other hand, the [110] and [001] directions are preferred directions for MA molecule in Pm-3m phase. We notice here that the P4mm structure was reported to be more stable than the Pm-3m (MA molecule lies along the [111] direction) with V ~ 250 Å^3^/f.u–260 Å^3^/f.u ^14^. We find that a rotation out of [111] direction of MA molecule further stabilizes the Pm-3m structure but the P4mm is still more stable than the Pm-3m structure (V < 256 Å^3^/f.u) with very small energy difference of 1–2 meV. Thus the preference for the MA orientation away from [111] plays an important role in selecting the structure.

Experimentally, the P4mm and Pm-3m structures are stable at room and higher temperature. On the other hand, our result at V < 265 Å^3^/f.u reveals that energy difference between P4mm and Pm-3m phases with different molecule orientations [111], [110], [001] is at maximum of 25 meV or equivalent to ~300 K. Such small energy differences easily allow the rotation of MA molecules in the cuboctahedral PbI_6_ by thermal excitation as is known. It is interesting to explore how the rotation of MA molecules will affect the properties of MAPbI_3_. Let start from cubic structure Pm-3m with a = b = c = 6.32 Å and MA molecule oriented along the [111] direction, point ${1}_{111}^{c}$, see Fig. 1. In principal, from the [111] direction MA molecule can rotate to any directions. To ease the study, we select two most symmetry directions: [110] and [001]. Our study shows that for the Pm-3m structure, the rotation of MA molecule from [111] direction to [110] or [001] will further stabilize the Pm-3m structure. It is noticed here that both Pm-3m structures with MA molecule oriented along [110] and [001] direction give almost the same total energy, E^C^[110] ~ E^C^[001], therefore we report only [110] direction. The conclusions for [001] case are the same for [110] direction. The rotation from point ${1}_{111}^{c}$ to point ${1}_{110}^{c}$ results in energy difference E(${1}_{110}^{c}$) − E(${1}_{111}^{c}$) = −16 meV ~ 186 °K. Our results show that at point ${1}_{110}^{c}$ the MAPbI_3_ structure is further stabilized from cubic Pm-3m to tetragonal P4mm structure by changing the lattice c-constant from 6.32 Å to 6.26 Å, point ${1}_{110}^{t}$, see Fig. 1, with E(${1}_{110}^{t}$) −E(${1}_{110}^{c}$) = −2 meV ~ 23 ^°^K. If the MA molecule is in [001] direction then the Pm-3m structure is further stabilized to P4mm structure by reducing the lattice constant from 6.32 Å to 6.30 Å, point ${1}_{001}^{t}$ with energy difference E(${1}_{001}^{t}$) −E(${1}_{001}^{c}$) = − 1meV ~ 11.6 ^°^K. In short, the rotation of MA molecule in different directions results in small energy differences in comparison to thermal energy at room temperature. Therefore MA molecules rotate in the cuboctahedral PbI_3_ under the thermal excitation near room temperature consistent with experiments. As discussed, such rotations are coupled to strain including volume, creating a breathing of the MAPbI_3_. In general, this type of coupling provides a local strain coupling that favors formatiion of regions or clusters of like orientation, and in the case of phase transitions, favors first order character with co-existence.

**Figure 2:**
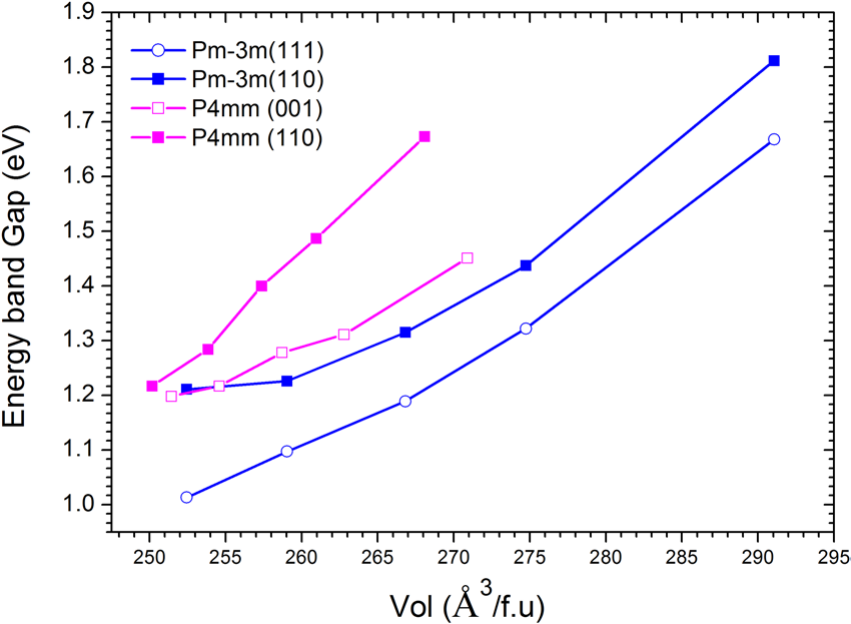
Energy band gap as a function of volume for Pm-3m and P4mm structure.

It is interesting to further explore the effect of such rotation on the electronic properties of MAPbI_3_. To investigate this effect we use the Tran-Blaha Modified Becke-Johnson (TB-MBJ) potential in general potential linearized augmented planewave (LAPW) method as implemented in the WIEN2k code^23,24^ to improve the value of band gap, which is under estimated by using the PBE^25^ or PBEsol^26^ GGA calculations. The TB-MBJ has been proved to give good band gap in comparison to GW method for s- and p –electron systems, which is applicable to MAPbI_3_ ^23^. Our results, see Fig. 1, show that the [110] direction is the most stable direction for MA molecule in both Pm-3m and P4mm structure. The rotation of MA molecule causes a large difference in band gap and in general MAPbI_3_ gets (i) *highest band gap with the tetragonal P4mm symmetry and MA molecule in [110] direction and* (ii) *lowest band gap with cubic Pm-*3*m symmetry and MA molecule in [111]*. Figure 1 shows that at V < 265 Å^3^/f.u the maximum energy difference between phases is about 22 meV~255 ^0^K which means that MA molecules are easy to rotate by thermal excitation at room temperature and higher. Such rotation not only changes the structure of MAPbI_3_ but also the energy band gap such as, at V = 252 Å^3^/f.u the rotation of MA molecule from [110] direction to [001] direction does not change much the energy band gap of MAPbI_3_, E_gap_ ~ 1.22 eV, while the rotation from [110] direction to [111] direction reduces the energy band gap to E_gap_ = 1.02 eV, 0.2 eV smaller. At higher volume the rotation of MA molecule results in larger band gap difference, larger shrinkage and expansion of volume. For example at V = 267 Å^3^/f.u the energy band gap of MAPbI_3_ is 1.2 eV in Pm-3m with molecule oriented in [111] direction. The rotation of molecule from [111] (E_gap_ ([111]) = 1.02 eV) to [110] results in band gap of 1.32 eV. When MA molecule is in [110] direction, a further deformation from Pm-3m structure to P4mm by reducing the lattice constant c results in volume reducing from V~ 267 Å^3^/f.u to V~257 Å^3^/f.u and the band gap gets value of 1.4 eV, see Fig. 2.

At lower volume V < 250 Å^3^/f.u the rotation of MA molecules in cuboctahedral PbI_3_ will transform the MAPbI_3_ from tetragonal P4mm to tetragonal I4/mcm^14^. Our calculations show that the transition results in big jump of energy band gap, such as at V = 252 Å^3^/f.u the P4mm has band gap of 1.22 eV while the I4/mcm at V = 251.02 Å^3^/f.u has energy band gap of 1.55 eV which is in agreement with experimental report^1^. Therefore at V ~ 251–252 Å^3^/f.u, the structure is sensitive with structural change and may co-exit three phases Pm-3m, P4mm and I4/mcm due to the rotation of molecules under thermal excitation. At lower volume V < 250 Å^3^/f.u the MAPbI_3_ is stabilized by I4/mcm structure due to larger energy difference between I4/mcm and P4mm phase and low thermal excitation energy.

**Figure 3:**
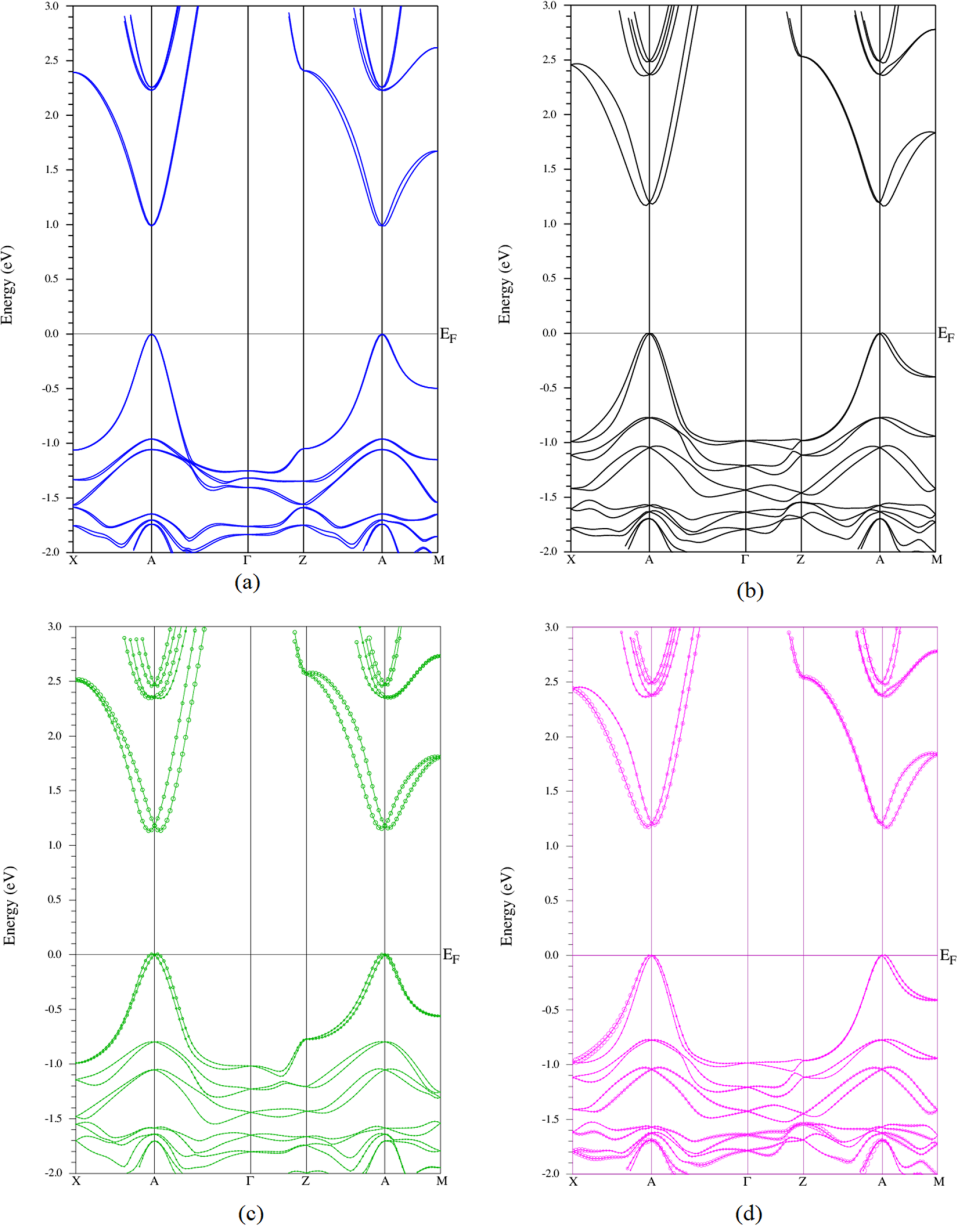
The band structure of MAPbI_3_ at: (**a**) points ${1}_{111}^{c}$, (**b**) ${1}_{110}^{c}$, (a = b = c = 6.32 Å), (**c**) ${1}_{110}^{t}$ (a = b = 6.32 Å c = 6.26 Å) and (**d**) ${1}_{001}^{t}$ (a = b = 6.32 Å, c = 6.30 Å) with X = [1/2 0 0] A [½ ½ ½], Γ [0 0 0], Z [0 0 ½] and M [½ ½ 0].

The rotation of MA molecule varies the energy band gap of MAPbI_3_ with an average amount of 0.2 eV down to the redshifted spectra, which help to enhance the efficiency of solar absorber. It is more insightful to know how such rotation will affect the band structure of MAPbI_3_. The band structures of MAPbI_3_ at points ${1}_{111}^{c}$, ${1}_{110}^{c}$,(a = b = c = 6.32 Å), ${1}_{110}^{t}$ (a = b = 6.32 Å c = 6.26 Å) and ${1}_{001}^{t}$ (a = b = 6.32 Å, c = 6.30 Å) are given in Fig. 3. The results show that for cubic structure when the MA molecule is in the [111] direction then the energy band gap is direct band gap at A[½ ½ ½]. The rotation of MA molecule out of the [111] direction results in indirect bandgaps and higher energy band gap for both Pm-3m and P4mm structure, see Fig. 3. The rotation of molecules therefore not only changes the crystal structure, tunes the band gap of MAPbI_3_ but also tunes the nature of the band gap from direct to indirect and vice versa.

The nature of the band gap (indirect vs. direct) comes from the momentum dependent splitting of energy bands by Rashba effect due to the spin-orbit coupling. This effect has been mainly studied for the I4/mcm structure and much less studied for cubic Pm-3m and tetragonal P4mm. In principle the spin-orbit coupling causes the energy band splitting in cubic Pm-3m and tetragonal P4mm symmetry but *there is no momentum dependent splitting of the energy band, the Rashba effect*. The momentum dependent splitting of energy bands that occurs in these structures (Fig. 3) is due to the rotation of MA molecules in the PbI_3_ cuboctahedral. This rotation lowers the symmetry. However, the fast rotation of MA molecules in PbI_3_ cuboctahedral at room and higher temperature in a time average would make the MA molecule behave as a point like particle (Fig. S1, supporting information). The effect of spin-orbit coupling on the momentum dependent splitting of energy band due to the rotation of MA molecules in PbI_3_ cuboctahedral is reported in supporting information. The study reveals a strong dependence of Rashba interaction coefficient on the volume and the orientation of MA molecule in PbI_3_ cuboctahedral_._

**Figure 4:**
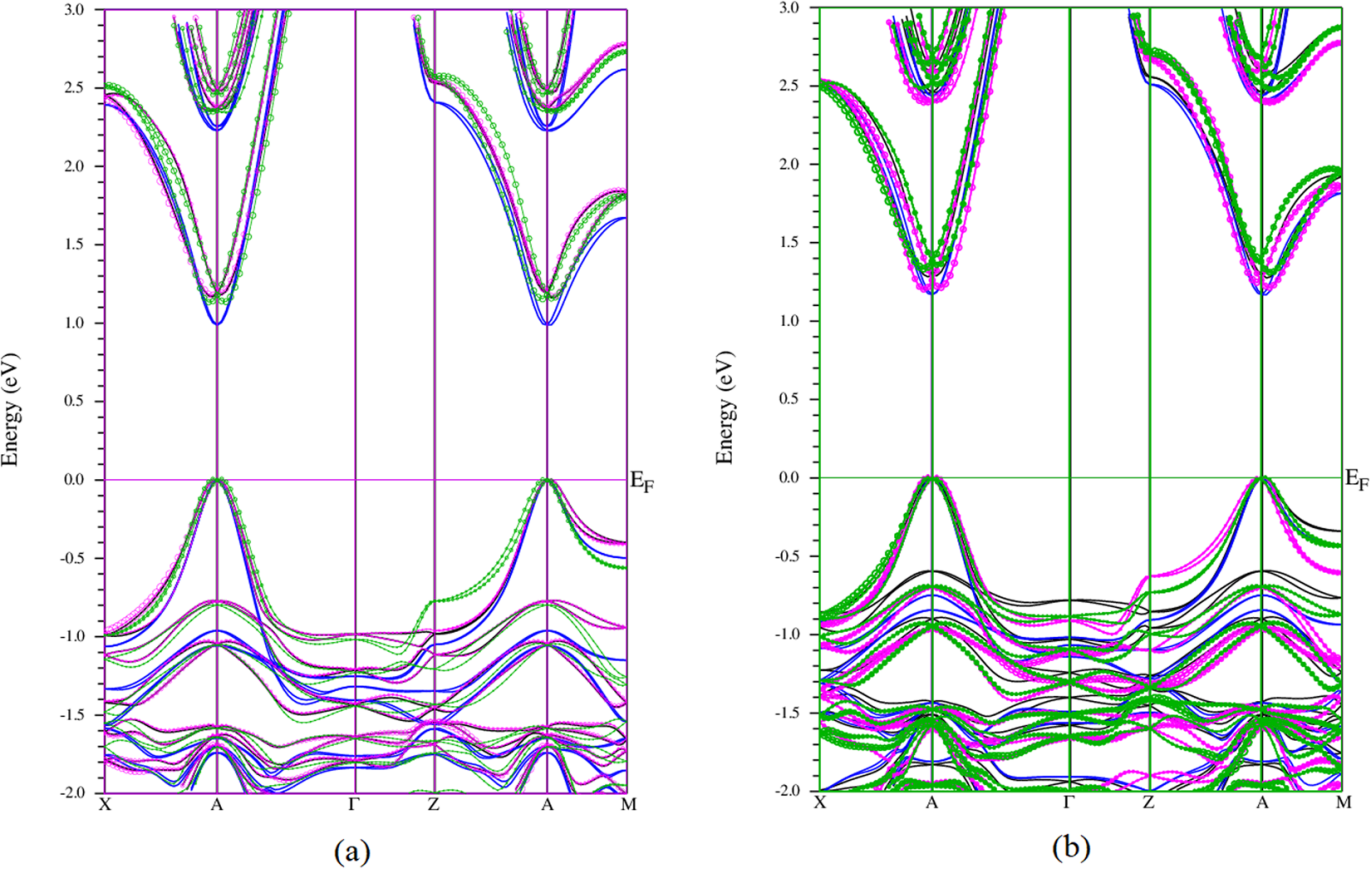
The new band structure of MAPbI_3_ which is the combination of 4 band structures in Fig. 3 (**a**) at a = b = 6.32 Å, see text for c-parameters. (**b**) The band structure with a = b = 6.438 Å and c = 6.438 Å(Pm-3m), c = 6.21 Å [110] (P4mm), c = 6.242 Å [001] (P4mm). The color lines/circles match with report in Fig. 3.

Since the rotational dynamics of the MA molecules may be correlated through strain coupling, [11] it may be useful to consider a new band structure, which is a combination of band structures of MAPbI_3_ with MA molecules in different directions. There are four structures in our cases: Pm-3m structure with MA molecule in [111] and [110]/[001] direction, P4mm structure with MA molecule in [110] and [001] direction, see Fig. 4. This is germane to the case where the correlation length for MA rotation is significantly longer than the unit cell dimension, so that local band structures are important. The new energy band structure has features of good solar absorber materials with *(1) multi energy bandgaps and (2) indirect energy bandgaps*. The most extension of energy band gap to the lower value comes from the rotation of MA molecule to the [111] direction. The multi energy band gap structure (1) allows more photons with different wavelengths are absorbed. The indirect energy band gap (2) prevents the recombination of electrons in the conduction band with holes in the valence band. *This mechanism enhances the electron density in the conduction band of MAPbI*_*3*_ *and their lifetime*.

**Figure 5:**
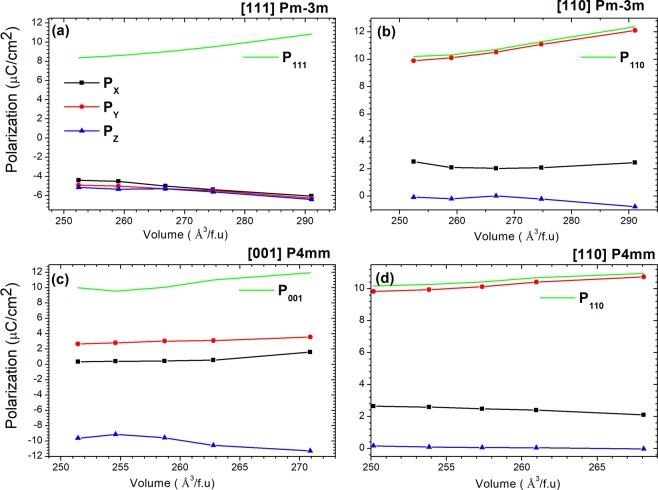
The Volume evolution of electron polarization of MAPbI3 with different MA orientations (**a**) cubic [111]; (**b**) cubic [110]; (**c**) P4mm [001]; and (**d**) P4mm [110].

The rotation of MA molecule in the cuboctaheral PbI_3_ causes an electronic polarization. The momentum dependent splitting of energy band only appears in case of anisotropy. To investigate this effect, we calculate the volume evolution of electronic polarization when the MA molecules are in different orientations. The results are shown in Fig. 5. The result clearly shows that for cubic structure when the MA molecule is in [111] direction then the polarizations in three different directions are almost the same. Such effect results in a very weak energy band splitting as shown in Fig. 3a. When MA molecule is in [110] or [001] direction, the electronic polarizations in different directions are very much different, see Fig. 5(b–d), causing a strong energy band splitting as shown in Fig. 3(b–d).

The rotation of MA molecule changes the lattice constants continuously in three directions from a to a-δ and vice versa with δ is the lattice constant difference between cubic Pm-3m and tetragonal P4mm when MA molecule is in the [110] direction. Since we are studying the MAPbI_3_ by using density functional theory i.e., one unit cell with periodic conditions, therefore this phenomenon is only valid when all molecules rotate at the same direction. In general, without any external constrain, at the same time all MA molecules in MAPbI_3_ rotate non-orientation. Such free rotation induces a continuous change of PbI_3_ cuboctahedral, shrink and expand in all directions. Therefore the lattice constant may be effectively considered as the quasi-cubic Pm-3m structure or quasi-tetragonal P4mm structure with lattice constants a = b~c.

## Conclusions

In summary, we have studied the impact of correlated orientations of MA molecules on the evolution of crystal structures, energy band structures and energy band splitting of MAPbI_3_ focusing on Pm-3m and P4mm structure. The results showed that at V = 250 Å^3^/f.u three structures I4/mcm, P4mm and Pm-3m coexist. The energy band gap of I4/mcm structure is 1.55 eV which is in perfect agreement with experimental report but it is about 1.2 eV for Pm-3m and P4mm structure when MA molecule is in the [001] and [110] direction. When MA molecule is in the [111] direction, the MAPbI_3_ is stabilized with Pm-3m structure with energy band gap of 1.02 eV. At higher volume the P4mm and Pm-3m is more stable than I4/mcm. Although the MAPbI_3_ is stable with Pm-3m structure and MA molecule is in [110] direction, the rotations of MA molecules induce the change of local lattice constants. Therefore MAPbI_3_ can exist in both Pm-3m and P4mm structures. The volume evolution of band structure shows an increasing trend of energy band gap with volume. The rotation of MA molecule defines the direct and indirect nature of energy band gap with an extension 0.2 eV of energy band gap down to redshifted region when MA molecule is in [111] direction. A new band structure has been proposed with new features *(1) multi energy bandgaps and (2) indirect energy bandgaps*. Study on the spin-orbit induced band splitting effect shows strong dependence of Rashba interaction coefficient on the volume and the orientation of MA molecule in cuboctahedral PbI_3_.

## Methods

For the calculations of electronic structures and related properties of MAPbI_3_ we use the projector augmented wave (PAW) method^27^ with the Perdew-Burke-Ernzerhof (PBE)^25^ and the PBE revised for solids (PBEsol)^26^ generalized gradient approximation (GGA) exchange correlation potentials as implemented in the VASP code^28^. The cut-off energy for the plane wave expansion of the wave functions is 500 eV, and all atoms in the unit cell are fully relaxed till the Hellman-Feynman forces are less than 0.005 eV/Å. The 6 × 6 × 6 Monkhorst-Pack grid of k-points^29^ for Brillouin zone integration was used in calculations for Pm-3m and P4mm structures. The semicore states of the Pb atoms are treated as valence electrons; i.e., 14 valence electrons for Pb (5d^10^6s^2^ 6p^2^). The I-5s^2^5p^5^, C-2s^2^2p^2^, N-2s^2^2p^3^ and H-1s were considered as valence electrons.

The symmetry of Pm-3m and P4mm is built based on the PbI_3_ frame without the presence of molecule CH_3_NH_3_. After the frame is built then the molecule CH_3_NH_3_ is added to the center of the PbI_3_ frame at different orientations [001], [110] and [111]. At each of specific orientation the MAPbI_3_ crystal structure is fully relaxed without any constrain on the symmetry. The obtained structures are therefore at P1 symmetry in general due to the presence of CH_3_NH_3_ molecule and they are very close to the Pm-3m or P4mm symmetry. Therefore they are called a pseudo-cubic Pm-3m or a pseudo-tetragonal P4mm.

We applied the Van der Waals correction force (vdW-DF2 or D2)^30^, which is proved to be the best in comparison the lattice constant of CH_3_NH_3_PbI_3_ with experimental data^31^, in our calculation and find that the PBEsol and PBE + vdWDF2 give the same results as reported by Menendex, ref.^31^. On the other hand, our results based on the vdW-D3 correction method by Grimme *et al*.^32^, which is reported by Thind *et al*.^33^, underestimate the lattice constant of the cubic structure Pm-3m in comparison to experimental data, see the table S1. Because of this we preferred to use PBEsol method instead of PBE-vdW-D3.

To calculate the energy band structure of MAPbI_3_ we use the WIEN2k software package^23^. This program allows to compute the electronic structure of MAPbI3 within DFT utilizing the full potential (linear) augmented plane wave + local orbitals (APW + lo) method and applying the MBJ method^24^. The atomic sphere radii of MAPbI_3_ are chosen as 2.5 a.u for Pb and I; 1.28 a.u for N, 1.34 a.u for C and 0.69 a.u for H. Inside the atomic spheres, the partial waves were expanded up to l_max_ = 10 and the number of plane waves was limited by a cut off K_max_ = 4.64 (a.u^−1^). The charge density was Fourier-expanded with G_max_ = 20 Ry. A k-mesh of 10 × 10 × 10 in the full Brillouin zone was used. In addition to the usual valence states, also extra local orbitals for “semi-core” states (Pb- 5b, 5d, 6 s, 6p; I-4d, 5 s, 5p; N: 2p, and C:2p) were added and considered as band states.

## Supplementary information

Multi Band Gap Electronic Structure in CH3NH3PbI3
