# Supplementary material for: Multi Band Gap Electronic Structure in CH3NH3PbI3
Source: Sci Rep. 2019 Feb 14;9:2144. doi: 10.1038/s41598-018-38023-2 (PMC6376135; doi:10.1038/s41598-018-38023-2)
Supplement: Supplementary file 1 — Multi Band Gap Electronic Structure in CH3NH3PbI3 [file 41598_2018_38023_MOESM1_ESM.docx]

Supporting information:

Multi Band Gap Electronic Structure In CH_3_NH_3_PbI_3_

*Khuong P. Ong,^1^ Shunnian Wu,^1^ Tien Hoa Nguyen,^2^ David J. Singh,^3^ Zhen Fan,^4^ Michael B. Sullivan,^1^ and Cuong Dang,^2^*

^1^ Institute of High Performance Computing, Agency of Science, Technology and Research (A*STAR), 1 Fusionopolis Way, 138632, Singapore

^2^ Centre for OptoElectronics and Biophotonics (COEB), School of Electrical and Electronic Engineering, The Photonics Institute (TPI), Nanyang Technological University Singapore, 50 Nanyang Avenue, 639798, Singapore

^3^ Department of Physics and Astronomy, University of Missouri, Columbia, MO 65211-7010 USA

^4^ Institute for Advanced Materials and Guangdong Provincial Key Laboratory of Optical Information Materials and Technology, South China Academy of Advanced Optoelectronics, South China Normal University, Guangzhou 510006, China

AUTHOR INFORMATION

Corresponding Author: Khuong P. Ong

*Email: ongpk@ihpc.a-star.edu.sg

Table S1. B-M equation fitted volume at minimum energy

|  | cubic |
| --- | --- |
| PBEsol | 252.8933 |
| PBE-vdW-D2 | 251.70 |
| PBE-vdW-D3 | 199.1863 |


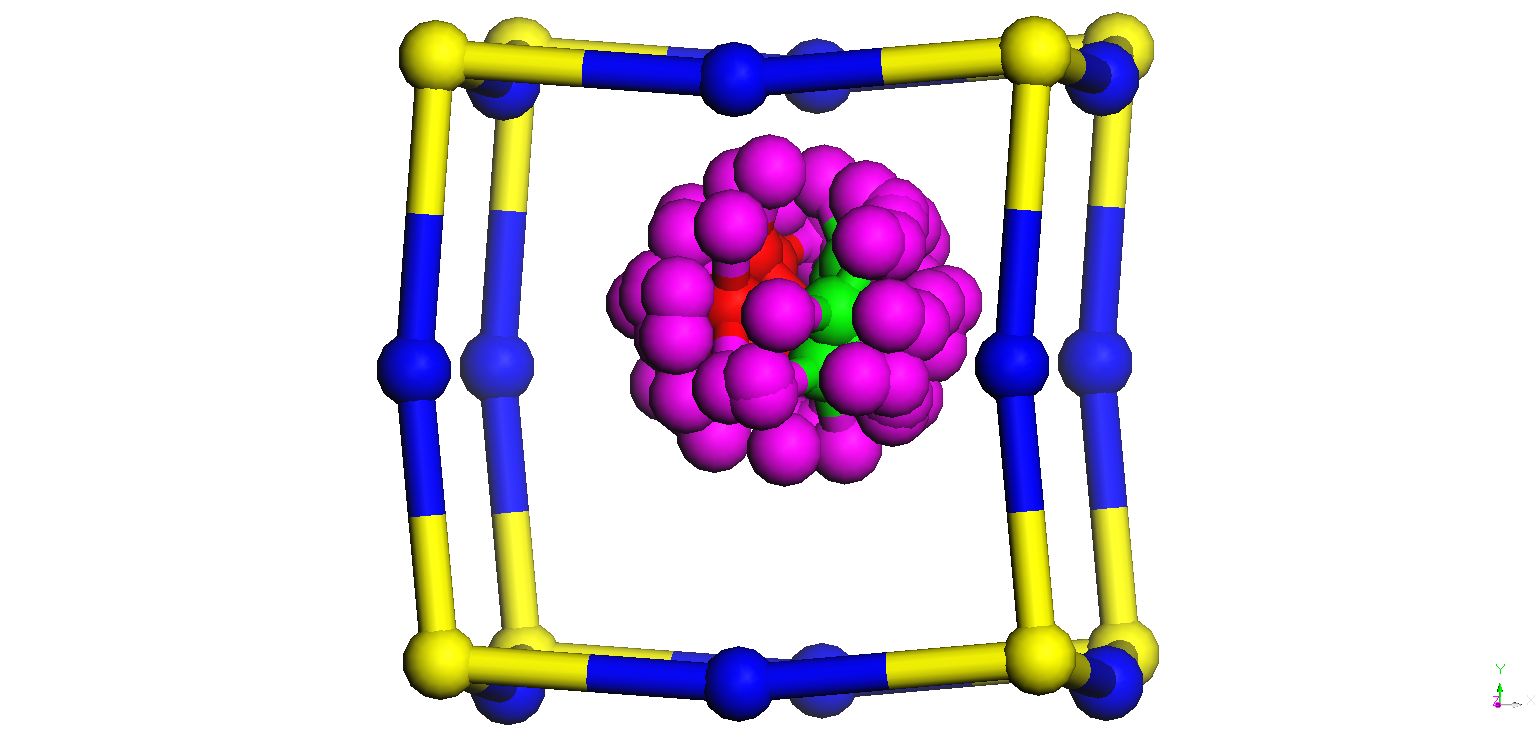


Fig.S1. Quasi molecule CH_3_NH_3_ in the cuboctahedral PbI_3_

To evaluate the effect of MA rotation to the splitting of the energy band structures we calculate the Rashba spin-orbit interaction coefficient which is given by $\alpha_{C}=\frac{{\Delta E}_{C}}{2{\Delta k}_{C}}$,^1^ here ΔE_C_ and Δk_C_ is the energy and momentum splitting in the conduction band due to the spin-orbit coupling (see Fig.S1. and Ref.[1] for details). Since the splitting in the valence band is small (see Fig.3) therefore we only evaluate the splitting in the conduction band.

Table S2. The Rashba coefficient of MAPbI_3_ in Pm-3m and P4mm structures

| Lattice constant ( Å) | $\alpha_{C}=\frac{{\Delta E}_{C}}{2{\Delta k}_{C}}$ [eV Å ^-1^] | | | |
| --- | --- | --- | --- | --- |
|  | AX | AΓ | AM | AZ |
| Pm-3m [110] |  |  |  |  |
| 6.320 | 1.45 | 1.24 | 1.66 |  |
| 6.375 | 1.27 | 1.23 | 1.49 |  |
| 6.438 | 1.01 | 0.94 | 1.29 |  |
| 6.500 | 0.97 | 0.91 | 1.07 |  |
| 6.627 | 0.75 | 0.50 | 0.71 |  |
| P4mm [001] |  |  |  |  |
| 6.320 | 1.79 | 1.80 | 1.35 | 1.47 |
| 6.375 | 1.74 | 1.72 | 1.37 | 1.25 |
| 6.438 | 1.86 | 1.75 | 1.66 | 1.03 |
| 6.500 | 1.66 | 1.53 | 1.88 | 0.82 |
| 6.627 | 1.73 | 1.69 | 2.00 | 0.44 |
| P4mm [110] |  |  |  |  |
| 6.320 | 1.51 | 0.94 | 1.59 |  |
| 6.375 | 1a.56 | 1.11 | 1.8 |  |
| 6.438 | 1.64 | 1.28 | 1.98 |  |
| 6.500 | 1.71 | 1.31 | 1.88 |  |
| 6.627 | 1.66 | 1.38 | 1.81 |  |

Volume evolution of the Rashba spin-orbit interaction coefficient in different directions AX, AΓ, AM and AZ when MA molecule is in different directions [001] and [110] is given in Fig.5. The results showed that for Pm-3m structure, the Rashba spin-orbit interaction coefficient decreases with the increase of volume in all directions of the energy splitting when MA molecule is in [110] or [001] directions. The deformation from Pm-3m structure to P4mm structure when MA molecule is in [110] direction increases the Rashba spin-orbit coefficient results in all directions but when MA molecule is in [001] direction the Rashba spin-orbit interaction coefficient in AX and AΓ direction does not change much and increase/decrease along the AM/AZ direction, respectively.


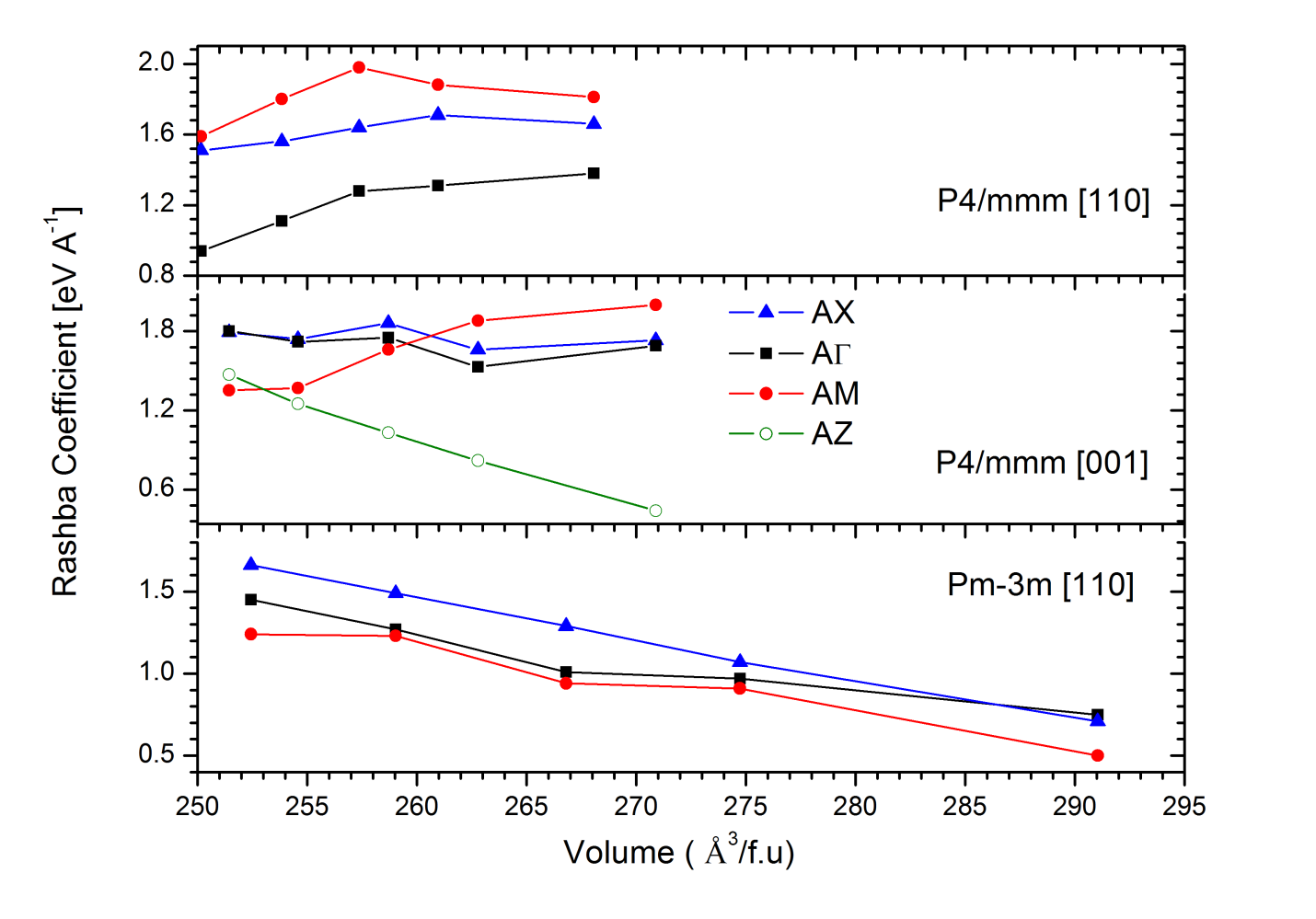


Fig.S2. Volume evolution of the Rashba spin- orbit interaction coefficient in different directions AX, AΓ, AM and AZ when MA molecule is in different directions [001] and [110].

**References**

1. T. Etienne, E. Mosconi, and F. D. Angelis, Dynamical Origin of the Rashba Effect in organohalide Lead Perovskites: A key to suppressed Carrier Recombination on Perovskite Solar cells, J. Phys. Chem. Lett. **7**, 1638 (2016)
